# Need Support and Regulatory Focus in Responding to COVID-19

**DOI:** 10.3389/fpsyg.2020.589446

**Published:** 2020-11-19

**Authors:** Leigh Ann Vaughn, Chase A. Garvey, Rachael D. Chalachan

**Affiliations:** Department of Psychology, Ithaca College, Ithaca, NY, United States

**Keywords:** need-support model, regulatory focus theory, self-determination theory, goals and motivation, self-regulation

## Abstract

Prevention focus is a self-regulatory orientation that serves the need for security, and promotion focus is a self-regulatory orientation that serves the need for growth. From mid-March to early April 2020, did people judge prevention focus to be more useful than promotion focus for responding to COVID-19? Our study tested and showed support for this hypothesis with 401 American and Canadian participants, who we sampled in 100-person waves on the first 4 Thursdays of the pandemic. For this study, we developed a new measure of the judged usefulness of promotion and prevention focus. Additionally, results showed that the judged usefulness of promotion and prevention focus related positively to support of the psychological needs for autonomy and relatedness, respectively, in responding to COVID-19. Exploratory analyses showed that day-to-day differences in autonomy, competence, and relatedness support and in promotion and prevention focus tended to be small, which is notable given the large-scale changes to social distancing, employment, and media coverage of the virus during this time. Our research could be useful for crafting persuasive advocacy and narrative communications that encourage social distancing to protect others about whom people care most.

## Introduction

In early March 2020, before the World Health Organization declared COVID-19 a pandemic, China and Italy had already issued widespread stay-at-home orders (World Health Organization, 2020a, b). That month, leaders in the United States and Canada also were encouraging people to socially distance, and widespread stay-at-home orders had begun in these countries (Hauck et al., 2020; Mervosh et al., 2020; Rev, 2020). Social distancing and following stay-at-home orders required being careful, exerting self-control, and doing what was expected: responsibilities deemed necessary for protecting oneself and others from the virus (Centers for Disease Control and Prevention, 2020c). According to regulatory focus theory, prevention focus is a self-regulatory orientation that serves the fundamental survival need of security (Higgins, 1997, 1998). It involves using vigilant strategies such as avoiding things that can be harmful (e.g., Freitas and Higgins, 2002) in order to protect the self and others, and fulfill responsibilities, duties, and obligations (for reviews, see Higgins, 1997, 1998; Molden et al., 2007; Scholer et al., 2019a). Regulatory focus theory proposes that promotion focus, in contrast, serves the fundamental survival need for growth (Higgins, 1997, 1998). It involves eagerly approaching things that are helpful (e.g., Freitas and Higgins, 2002) to fulfill hopes and aspirations. Regulatory focus theory has been applied to many outcomes pertaining to judgment, decision-making, and information processing in many domains such as health, relationships, work, and education (for reviews, see Higgins, 1997, 1998; Molden et al., 2007; Ludolph and Schulz, 2015; Scholer et al., 2019a). The current research appears to be the first to examine regulatory focus in the context of responding to COVID-19, and we predicted that participants would judge prevention focus to be more useful than promotion focus for responding to the virus.

The extraordinary circumstances caused by the COVID-19 pandemic offered a unique opportunity to test hypotheses about regulatory focus and its relationship with the three fundamental psychological needs proposed by self-determination theory: autonomy, competence, and relatedness (Deci and Ryan, 2000; Ryan and Deci, 2017). Autonomy-supportive circumstances help one do what one really wants to. Competence-supportive circumstances help one feel capable of taking on and mastering hard problems. Relatedness-supportive circumstances help one feel close and connected to others. Self-determination theory proposes that these three needs are important for psychological well-being and optimal motivation for long-term goal pursuit, and a large body of research supports this hypothesis in areas such as health, close relationships, work, school, the arts, and sport (for reviews, see Deci and Ryan, 2000, 2008; Ryan and Deci, 2008, 2017; Vansteenkiste et al., 2020).

The hypotheses we tested about relationships between regulatory focus and psychological need support came from the need support model (Vaughn, 2017), which bridges regulatory focus theory (Higgins, 1997, 1998) and self-determination theory (Deci and Ryan, 2000; Ryan and Deci, 2017). This model proposes that when people are in a promotion focus, they are motivated to view their circumstances in ways that encourage eagerness—that is, as being more supportive of autonomy, competence, and relatedness (Vaughn, 2017). These relationships can occur because eagerness helps individuals in a promotion focus feel that what they are doing is valuable and motivating (e.g., Higgins, 2000, 2005). Additionally, when people view their circumstances as more need-supportive, they are more likely to become promotion-focused to capitalize on opportunities for growth. Research on the need-support model corroborates this hypothesis especially strongly for autonomy support (Vaughn, 2017, 2019; Vaughn et al., 2020; also see Kim et al., 2019). The positive relationship between promotion focus and autonomy support may occur, in part, because promotion focus often involves viewing goals as hopes and aspirations, and autonomy support involves viewing circumstances as providing opportunities to pursue what one ideally would like to do (e.g., Vaughn, 2018, 2019; Vaughn et al., 2020).

The need-support model also proposes that when people are in a prevention focus, they are motivated to view their circumstances in ways that encourage vigilance—specifically, as being less supportive of needs for autonomy, competence, and relatedness (Vaughn, 2017). These relationships can occur because vigilance helps individuals in a prevention focus to feel that what they are doing is valuable and motivating (e.g., Higgins, 2000). Conversely, when people view their circumstances as less need-supportive, they are more likely to become prevention focused to maintain the good things they have. Research on the need-support model corroborates this hypothesis particularly for autonomy and relatedness support (Vaughn, 2017, 2019; Vaughn et al., 2020). The finding that prevention focus associates negatively with autonomy and relatedness support may occur, in part, because prevention focus involves viewing goals as duties and “oughts,” which people often view as not very autonomy supportive (e.g., Deci and Ryan, 2000; Koestner et al., 2002; Milyavskaya et al., 2014; Chen et al., 2015; Ryan and Deci, 2017; Vansteenkiste et al., 2020). Fulfilling duties and obligations may also be especially important for maintaining relationships with people to whom one does not feel close (e.g., Vaughn, 2018, 2019). Research on the need-support model has also shown that prevention-focused experiences often are high in competence support compared to experiences without any specific need support, which may occur because people need to feel competent if they are to self-regulate (Vaughn, 2017, 2019; Vaughn et al., 2020).

Earlier research testing the need-support model used retrospective reports on everyday types of activities (Vaughn, 2017, 2019; Kim et al., 2019; Vaughn et al., 2020) and performance tasks in controlled experiments (Vaughn, 2017). We based hypotheses about need support and regulatory focus in responding to COVID-19 on this earlier research (especially Vaughn, 2017, Study 2):

• When controlling for relationships between the types of need support, autonomy support will relate significantly and positively to promotion focus.
• When controlling for relationships between the types of need support, autonomy and relatedness support will relate significantly and negatively with prevention focus, and competence support will relate significantly and positively with prevention focus.

These hypotheses were tentative for several reasons. One is that responding to COVID-19 is different from any personal experience or experimental task studied in earlier research. Another is that we examined judgments of current need support and usefulness of promotion and prevention focus rather than retrospective reports of need support and regulatory focus (c.f., Vaughn, 2017, 2019; Kim et al., 2019; Vaughn et al., 2020). We assessed prospective rather than retrospective regulatory focus because when we were designing the study, many people in the United States and Canada had not yet taken many actions to protect themselves or others from COVID-19.

Thus, to test hypotheses about the judged usefulness of promotion and prevention focus for responding to COVID-19, we developed a new measure. It included items about attention to hopes/ideals and duties/oughts, which are the most common ways to operationally define promotion and prevention focus (e.g., Summerville and Roese, 2008; Hodis, 2017). We also based items on research about regulatory focus and openness to new experiences (Vaughn et al., 2008), how regulatory focus relates to episodes of exploration and self-control (Manczak et al., 2014; Vaughn et al., 2020), and questionnaire measures of chronic and situational regulatory focus (Higgins et al., 2001; Lockwood et al., 2002; Ouschan et al., 2007; Wallace et al., 2009; Haws et al., 2010; Fay et al., 2019). We expected that participants would judge prevention focus to be more useful than promotion focus for responding to COVID-19, and our test of this hypothesis served as a test of the validity of our new measure.

The current research took place on the first 4 Thursdays of the COVID-19 pandemic (March 12, 19, 26, and April 2, 2020), and each day of data collection served as a check on the replicability of the results on the other days. We took a different sample of 100 participants on each day, which meant that any differences between days of the study could reflect the degree of virus spread, messages about how to respond, impacts on employment and relationships, and other confounded factors. Given the widespread shutdowns and messaging from leaders during this time (Hauck et al., 2020; Mervosh et al., 2020; Rev, 2020), finding no differences in need support or regulatory focus would be surprising and noteworthy. We expected that if there were between-week differences in need support and subjective usefulness of promotion and prevention focus, they might correspond to some degree with the start of widespread stay-at-home orders. Such orders could reduce people’s sense of choice and subjective competence in how to respond to COVID-19, as well as their sense of feeling close and connected to others when responding to the virus. If so, there could be lower support for autonomy, competence, and relatedness in responding to COVID-19 as the study went on. Judged usefulness of prevention focus for responding to COVID-19 could increase if the pandemic touched more people’s lives directly over time, which could go along with lower judged usefulness of promotion focus for responding to the virus. Because no one knew in advance what would happen over the first 4 Thursdays of the pandemic, our tests of differences between days of the study were exploratory and interpretation of such differences remain tentative for the purpose of hypothesis generation. Our questions were:

• What actions did participants take most to deal with COVID-19, and what differences were there across weeks of the study?
• How did need support and judged usefulness of promotion and prevention in responding to COVID-19 differ between the days of the study?

## Materials and Methods

We collected data from 100 different participants on each of the first 4 Thursdays of the pandemic, to ensure that we had the same sized sample each time. Participants resided in the United States and Canada.

### Reporting

Approval was obtained from the ethics committee of Ithaca College. The procedures used in this study adhere to the tenets of the Declaration of Helsinki. We report how we determined our sample size, as well as all data exclusions, all manipulations, and all measures in the study. This study was not preregistered. For data analyses, we used SPSS 26, apaTables (Stanley and Spence, 2018), and jamovi (The jamovi project, 2019, Version 1.1.9). The data files, data dictionaries, and materials for the current investigation are available at https://osf.io/8ek2w/. We conducted sensitivity power analyses with G^∗^Power (Faul et al., 2007), and the results of these power analyses are in the relevant parts of the results section.

### Participants and Recruiting

The target sample size was 400 participants, based on available research funds. We recruited participants through Prolific, where we set the criteria for participation. Participants had to be at least 18 years old, live in the United States or Canada, and have English as their first language. They also had to have an acceptance rate on Prolific studies of at least 95%, and to have not done any of our lab’s prior studies on Prolific. To reduce variability in written responses, they had to do the study on a tablet or desktop computer rather than a phone. The study took approximately 8 min, so respondents received USD $0.88 for participating.

Our goal was to collect data from 100-person subsamples on 4 days during the first 4 weeks of the COVID-19 pandemic: March 12 (the day after the World Health Organization declared the pandemic), March 19, March 26, and April 2. Data collection on these dates occurred between noon and 4:00 p.m., Eastern Standard Time. Two participants on March 19 were excluded because they provided written responses that were not fluent or did not make sense, and we replaced them on that day. One participant on April 2 was replaced by Prolific, but they provided complete data, so we compensated this participant and used their data.

In the final sample of 401 participants, 286 (71.3%) resided in the United States, and 211 (52.6%) identified as female.^1^ Mean age was 32.4 years. Participants selected the racial and ethnic categories to which they belonged; 343 selected White (85.5%), 33 selected Asian (8.2%), 27 selected Black or African American (6.7%), 19 selected Hispanic or Latinx (4.7%), four selected Native American or Alaska Native (1%), and three selected “other” (0.7%). The methodology and data files at https://osf.io/8ek2w/contain the other background information we collected, including education, occupation, and state/province/territory of residence.

### Materials

#### Writing Task

The first page of stimulus materials was titled “Your Personal Responses to the COVID-19 Pandemic.” It stated, “First, we would like to learn about how you personally are responding to the COVID-19 coronavirus pandemic. This is a general question, and you can write about your thoughts, feelings, and/or behaviors. Please take a minute or two and write about your responses to this pandemic.”

#### Need Support

The second page of stimulus materials automatically piped in what the participants wrote on the first page and asked them to rate how much they agreed with 18 statements about their responses to the COVID-19 pandemic (1 = *strongly disagree*, 7 = *strongly agree*). These statements were the Balanced Measure of Psychological Needs (BMPN; Sheldon and Hilpert, 2012), which contains six-item subscales that measure support for autonomy (e.g., “I am really doing what interests me,” “There are people telling me what I have to do”; reverse-scored), competence (e.g., “I take on and master hard challenges,” “I do stupid things that make me feel incompetent”; reverse-scored), and relatedness (e.g., “I feel close and connected with other people who are important to me,” “I feel unappreciated by one or more important people”; reverse-scored). After appropriate reverse scoring, we calculated an index for each subscale by taking the mean of the relevant items. Table 1 shows the Cronbach’s alphas for these indexes.

**TABLE 1:** Cronbach’s alphas, means, standard deviations, and correlations.

Day and variable	Cronbach’s α	*M*	*SD*	1	2	3	4
**All Thursdays combined (*N* = 401)**							
1. Autonomy	0.70	4.03	1.03				
2. Competence	0.80	4.78	1.06	0.46**			
3. Relatedness	0.74	4.94	1.04	0.53**	0.60**		
4. Promotion	0.77	3.68	1.25	0.29**	0.12*	0.14**	
5. Prevention	0.74	5.60	0.86	0.06	0.12*	0.25**	0.03
**March 12 (*N* = 100)**							
1. Autonomy	0.68	4.46	0.96				
2. Competence	0.79	4.71	1.05	0.60**			
3. Relatedness	0.74	5.04	1.06	0.59**	0.66**		
4. Promotion	0.77	3.62	1.08	0.24*	0.16	0.13	
5. Prevention	0.67	5.36	0.79	0.14	0.19	0.21*	0.05
**March 19 (*N* = 100)**							
1. Autonomy	0.70	3.99	1.03				
2. Competence	0.76	4.83	1.00	0.54**			
3. Relatedness	0.76	5.07	1.04	0.47**	0.64**		
4. Promotion	0.80	3.52	1.33	0.34**	0.02	0.12	
5. Prevention	0.72	5.85	0.79	0.06	0.19	0.29**	0.04
**March 26 (*N* = 100)**							
1. Autonomy	0.66	3.89	0.95				
2. Competence	0.83	4.77	1.12	0.43**			
3. Relatedness	0.72	4.81	1.01	0.46**	0.60**		
4. Promotion	0.76	3.73	1.22	0.22*	0.15	0.06	
5. Prevention	0.75	5.48	0.87	0.15	0.00	0.21*	−0.05
**April 2 (*N* = 101)**							
1. Autonomy	0.75	3.79	1.07				
2. Competence	0.80	4.81	1.08	0.38**			
3. Relatedness	0.74	4.85	1.05	0.58**	0.53**		
4. Promotion	0.76	3.87	1.34	0.42**	0.15	0.28**	
5. Prevention	0.78	5.73	0.89	0.06	0.10	0.32**	0.08

M and SD represent mean and standard deviation, respectively. * indicates p < 0.05. ** indicates p < 0.01.

#### Judged Usefulness of Promotion and Prevention

The third page of stimulus materials automatically piped in what the participants wrote on the first page. It asked participants to “Please indicate how much each of the following would support or impair how you respond to the COVID-19 pandemic.” Participants responded on a 7-point scale (1 = strongly impair, 4 = neither impair nor support, 7 = strongly support). Five items represented promotion (e.g., “Being spontaneous”) and five represented prevention (e.g., “Exerting self-control”). Table 2 shows these items.

**TABLE 2:** Communalities and factor loadings from the exploratory factor analysis on usefulness of promotion and prevention focus.

	Factor		Communalities	
Item	1	2	Initial	Extracted
6. Being spontaneous	**0.769**	−0.021	0.456	0.589
8. Not missing out on anything good	**0.724**	−0.109	0.429	0.525
10. Doing what I would ideally like to	**0.649**	−0.019	0.347	0.420
2. Trying new things just because they could be interesting	**0.577**	0.030	0.290	0.337
4. Being enthusiastic^a^	**0.425**	**0.447**	0.361	0.407
3. Exerting self-control	−0.043	**0.723**	0.411	0.520
5. Fulfilling my duties and obligations	0.249	**0.638**	0.451	0.491
7. Doing what is expected of me	0.129	**0.602**	0.394	0.391
9. Being careful	−0.199	**0.606**	0.311	0.390
1. Not making mistakes	−0.156	**0.492**	0.222	0.255

*N = 401. Maximum likelihood exploratory factor analysis and direct oblimin rotation with delta = 0. Loadings are from the pattern matrix, and loadings over 0.40 are in bold font. Factor 1 represents judged usefulness of promotion focus, and Factor 2 represents judged usefulness of prevention focus. ^*a*^This item unexpectedly loaded on both factors and was not retained in the promotion and prevention indexes, to maximize ease of interpreting results with these indexes.*

We submitted the 10 judged usefulness items to an exploratory factor analysis using maximum likelihood estimation and direct oblimin rotation, with delta = 0. The Kaiser-Meyer-Olkin measure showed that the sampling was adequate, KMO = 0.781. Bartlett’s test of sphericity showed that the correlation structure was adequate for analyses, χ^2^(45) = 1122.02, *p* < 0.001. Table 1 shows the pattern-matrix factor loadings and the communalities for the items. These factors together accounted for 43.25% of the variance, and the promotion and prevention factors correlated at *r* = 0.072. Each item loaded > 0.40 on only one factor, except for “Being enthusiastic.” We had expected the enthusiasm item and the other four promotion items to load only on the promotion factor, and five items to load on the prevention factor.

To maximize ease of interpreting the results with the promotion and prevention measures in this study, we did not include “Being enthusiastic” in either the promotion index or the prevention index. Instead, we analyzed this item separately, as described below. When we re-ran the factor analysis without this item, KMO = 0.755 and χ^2^(36) = 945.26, *p* < 0.001. The resulting promotion and prevention factors accounted for 43.61% of the variance, and they correlated at *r* = −0.003.^2^ Table 2 shows the Cronbach’s alphas for the final promotion and prevention indexes and the need-support indexes.

#### Actions Already Taken

The fourth page of stimulus materials asked participants to indicate (yes or no) which of 20 actions they had already taken to respond to the COVID-19 pandemic. We got these actions from looking at web pages on this topic in early March (Centers for Disease Control and Prevention, 2020b; Reinstein, 2020; Ries, 2020) and choosing actions that did not assume that someone in the home was already sick. Table 6 shows these actions.^3^ The actions did not include wearing a face mask, because in March 2020, organizations such as the World Health Organization did not recommend this for the general public (e.g., Lacina, 2020).

## Results

After providing descriptive statistics and correlations between need support and regulatory focus, we describe the analyses that tested our hypotheses. Then we describe the exploratory analyses of differences between weeks of the study. Because of the large number of results, we provide most of the statistics in tables. We describe sensitivity power analyses in footnotes to make it easier to follow the main results.

### Descriptive Statistics and Correlations for Need Support and Regulatory Focus

Table 2 shows the Cronbach’s alphas and descriptive statistics for the measures of need support and usefulness of promotion and prevention focus, as well as correlations between these measures. This table displays results for the entire sample and for each week. Support for autonomy, competence, and relatedness tended to correlate strongly (as in other research, e.g., Vaughn, 2017). The strongest correlations with promotion were with autonomy support, and the strongest correlations with prevention were with relatedness support.^4^

### Tests of Hypotheses

#### Relative Usefulness of Promotion and Prevention Focus

**FIGURE 1:**
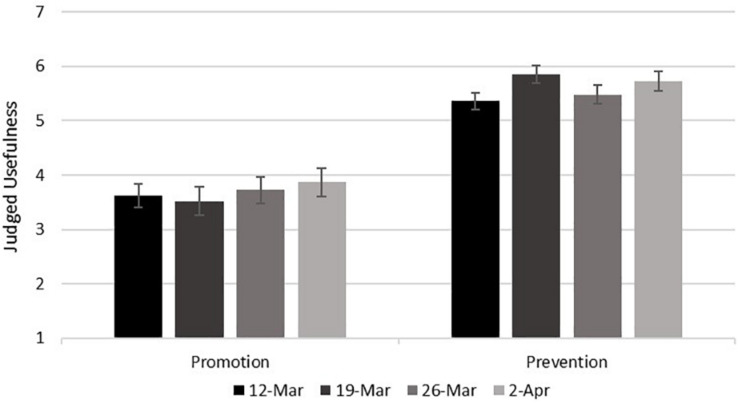
Judged usefulness of promotion and prevention focus for responding to COVID-19 as a function of the day of the study. Error bars represent 95% confidence intervals.

We expected that participants would judge prevention focus to be more useful than promotion focus for responding to COVID-19. As shown in Table 3 and Figure 1, this hypothesis was supported. The differences between promotion and prevention in the paired-samples *t*-tests were very large, both overall and within each day of the study (*d*s > 1.10).^5^

**TABLE 3:** Tests of differences between judged usefulness of promotion and prevention focus for responding to COVID-19.

Day	*t*	*df*	*p*	Mean diff.	*SD* diff.	95% CI	*d*
All Thursdays combined	25.81	400	<0.001	1.92	1.49	[1.77, 2.07]	1.29
March 12	13.38	99	<0.001	1.75	1.31	[1.49, 2.01]	1.34
March 19	15.37	99	<0.001	2.33	1.52	[2.03, 2.63]	1.54
March 26	11.45	99	<0.001	1.76	1.53	[1.45, 2.06]	1.14
April 2	12.09	100	<0.001	1.85	1.54	[1.55, 2.16]	1.20

*Positive numbers indicate higher scores for prevention. CI, confidence interval; d, Cohen’s d.*

#### Regulatory Focus as a Function of Need Support

As expected, autonomy support in responding to COVID-19 was the only significant predictor of the judged usefulness of promotion focus for responding to the virus, when accounting for relationships between the types of need support and the usefulness of prevention focus.^6^ This relationship was statistically significant in the total sample and in each day of data collection except March 12. Also as expected, this relationship was positive, both overall and within each week of the study. Table 4 shows the results of the multiple regression analyses on the promotion measure.

**TABLE 4:** Multiple regressions modeling relationships between need support and usefulness of promotion.

Day and predictor	*B*	β	*sr*^2^	*p*	95% CI for *B*
**All Thursdays combined**					
**Autonomy**	**0.36**	**0.30**	**0.06**	**< 0.001**	**[0.22, 0.50]**
Competence	−0.01	−0.01	0.00	0.872	[−0.15, 0.13]
Relatedness	−0.02	−0.02	0.00	0.806	[−0.18, 0.14]
Usefulness of prevention	0.02	0.02	0.00	0.743	[−0.12, 0.17]
**March 12**					
Autonomy	0.27	0.24	0.03	0.075	[−0.03, 0.56]
Competence	0.04	0.04	0.00	0.775	[−0.24, 0.33]
Relatedness	−0.04	−0.04	0.00	0.787	[−0.32, 0.24]
Usefulness of prevention	0.02	0.01	0.00	0.901	[−0.26, 0.29]
**March 19**					
**Autonomy**	**0.58**	**0.45**	**0.14**	**< 0.001**	**[0.28, 0.87]**
Competence	−0.36	−0.27	0.04	0.040	[−0.71, −0.02]
Relatedness	0.09	0.07	0.00	0.571	[−0.23, 0.42]
Usefulness of prevention	0.07	0.04	0.00	0.651	[−0.25, 0.40]
**March 26**					
**Autonomy**	**0.30**	**0.23**	**0.04**	**0.047**	**[0.00, 0.59]**
Competence	0.13	0.11	0.01	0.377	[−0.15, 0.40]
Relatedness	−0.13	−0.11	0.01	0.424	[−0.45, 0.19]
Usefulness of prevention	−0.09	−0.06	0.00	0.549	[−0.37, 0.20]
**April 2**					
**Autonomy**	**0.50**	**0.40**	**0.10**	**0.001**	**[0.21, 0.79]**
Competence	−0.04	−0.03	0.00	0.763	[−0.31, 0.23]
Relatedness	0.06	0.05	0.00	0.703	[−0.27, 0.40]
Usefulness of prevention	0.07	0.05	0.00	0.646	[−0.23, 0.37]

*B, unstandardized regression weights; β, standardized regression weights; sr^2^, semi-partial correlation squared; CI, confidence interval. Bold font indicates rows with significant effects.*

Unexpectedly, the only significant predictor of the judged usefulness of prevention focus for responding to COVID-19 was relatedness support, and the relationship was positive rather than negative. This relationship was statistically significant in the total sample and in each day of data collection except March 12. We had expected that each type of need support could be a significant predictor of the judged usefulness of prevention focus for responding to COVID-19. Specifically, we expected that the relationships with autonomy and relatedness support would be negative, and the relationship with competence support would be positive, both overall and within each week of the study. Table 5 shows the results of these multiple regression analyses on the prevention measure.^7^

**TABLE 5:** Multiple regressions modeling relationships between need support and usefulness of prevention.

Day and predictor	*B*	β	*sr*^2^	*p*	95% CI for *B*
**All Thursdays combined**					
Autonomy	−0.08	−0.10	0.01	0.097	[−0.18, 0.01]
Competence	−0.02	−0.03	0.00	0.629	[−0.12, 0.07]
**Relatedness**	**0.26**	**0.32**	**0.06**	**< 0.001**	**[0.16, 0.37]**
Usefulness of promotion	0.01	0.02	0.00	0.743	[−0.06, 0.08]
**March 12**					
Autonomy	−0.00	−0.00	0.00	0.990	[−0.22, 0.22]
Competence	0.06	0.08	0.00	0.584	[−0.15, 0.27]
Relatedness	0.12	0.16	0.01	0.254	[−0.09, 0.33]
Usefulness of promotion	0.01	0.01	0.00	0.901	[−0.14, 0.16]
**March 19**					
Autonomy	−0.10	−0.13	0.01	0.307	[−0.30, 0.09]
Competence	0.05	0.06	0.00	0.657	[−0.17, 0.27]
**Relatedness**	**0.23**	**0.30**	**0.05**	**0.022**	**[0.03, 0.43]**
Usefulness of promotion	0.03	0.05	0.00	0.651	[−0.10, 0.16]
**March 26**					
Autonomy	0.12	0.13	0.01	0.277	[−0.09, 0.33]
Competence	−0.16	−0.21	0.03	0.100	[−0.36, 0.03]
**Relatedness**	**0.24**	**0.28**	**0.05**	**0.031**	**[0.02, 0.47]**
Usefulness of promotion	−0.04	−0.06	0.00	0.549	[−0.19, 0.10]
**April 2**					
Autonomy	−0.17	−0.20	0.02	0.106	[−0.37, 0.04]
Competence	−0.06	−0.08	0.00	0.498	[−0.25, 0.12]
**Relatedness**	**0.39**	**0.47**	**0.12**	**< 0.001**	**[0.18, 0.61]**
Usefulness of promotion	0.03	0.05	0.00	0.646	[−0.11, 0.17]

*B, unstandardized regression weights; β, standardized regression weights; sr^2^, semi-partial correlation squared; CI, confidence interval. Bold font indicates rows with significant effects.*

### Exploratory Analyses

#### How the Enthusiasm Item Related to Need Support

Because the enthusiasm item unexpectedly loaded on both the promotion and prevention factors, we examined whether it related both to autonomy support (like the promotion index) and to relatedness support (like the prevention index). In an exploratory regression analysis with the combined sample, we treated the autonomy, competence, and relatedness as predictors of the enthusiasm item. Relatedness was the strongest significant predictor: *B* = 0.28, 95% CI for *B* [0.12, 0.44], β = 0.22, *p* < 0.001, *sr*^2^ = 0.03. Autonomy also was a significant predictor: *B* = 0.17, 95% CI for *B* [0.03, 0.31], β = 0.13, *p* = 0.019, *sr*^2^ = 0.01. Competence was not a significant predictor: *B* = 0.02, 95% CI for *B* [−0.13, 0.16], β = 0.01, *p* = 0.827, *sr*^2^ < 0.01. We limited this exploratory analysis to the combined sample because we wanted to maximize statistical power to predict this single-item dependent variable.

#### Differences in Actions Taken to Respond to COVID-19 Between Days of the Study

To learn about participants’ responses to COVID-19 during the Thursdays of the study, we asked which of 20 actions they had already taken. The results of chi-square analyses on responses (yes vs. no) by day of the study are in Table 6. These actions are in order of effect size, while the order of the items in the questionnaire is indicated by number. Four actions showed very large differences across the days of the study: self-quarantining, not gathering in public places, limiting close contact with others (about 6 feet), and stocking up on groceries. All 20 actions were endorsed more on March 19 than on March 12. Few actions were endorsed more on March 26 than on March 19, and any differences were relatively small. Fifteen actions were endorsed more on April 2 than on March 26, but these differences also were relatively small.^8^

**TABLE 6:** Actions that participants (out of 100 participants each day^a^) had already taken to respond to Covid-19, as a function of the day of the study.

	Day of study				Differences between days		
Action	March 12	March 19	March 26	April 2	Pearson chi-square	*p*	Cramér’s V
10. Self-quarantining	20	74	80	86	120.22	<0.001	0.548
14. Not gathering in public places	61	99	99	97	103.77	<0.001	0.509
18. Limiting close contact with others (about 6 feet)	45	87	90	98	103.40	<0.001	0.508
2. Stocking up on groceries	30	79	85	86	101.46	<0.001	0.503
9. Providing support to others	47	74	73	81	30.21	<0.001	0.274
8. Reaching out to others for support	26	54	56	59	27.70	<0.001	0.263
17. Staying away from others who are sick	83	97	94	98	19.85	<0.001	0.222
3. Stocking up on medicine	24	49	46	31	18.52	<0.001	0.215
15. Talking with supervisors or teachers about work that can be done from home	39	68	52	60	18.28	<0.001	0.213
16. Identifying aid organizations in your community	11	33	29	30	15.53	0.001	0.197
4. Checking in with work and school about closures	65	87	77	82	14.96	0.002	0.193
6. Figuring out how to work from home	55	73	72	76	12.13	0.007	0.174
11. Talking with your neighbors about emergency planning	7	18	6	9	10.04	0.018	0.158
1. Buying soap and disinfectants	59	77	73	76	9.84	0.020	0.157
19. Cleaning frequently touched surfaces and objects daily with household detergent and water	50	68	67	68	9.83	0.020	0.157
13. Keeping track of school dismissals in your community	44	58	41	45	6.91	0.075	0.131
12. Creating an emergency contact list	8	18	11	8	6.76	0.080	0.130
7. Washing your hands regularly	95	99	99	100	6.15	0.104	0.124
5. Paying attention to local news	89	97	94	94	5.21	0.157	0.114
20. Covering your coughs and sneezes with a tissue	83	87	89	87	1.58	0.664	0.063

*^*a*^On each day, N = 100 except on April 2, when it was 101. Degrees of freedom = 3. Actions are in order of effect size, with the order of the items in the questionnaire indicated by item numbers.*

#### Differences in Need Support and Regulatory Focus Between Days of the Study

**FIGURE 2:**
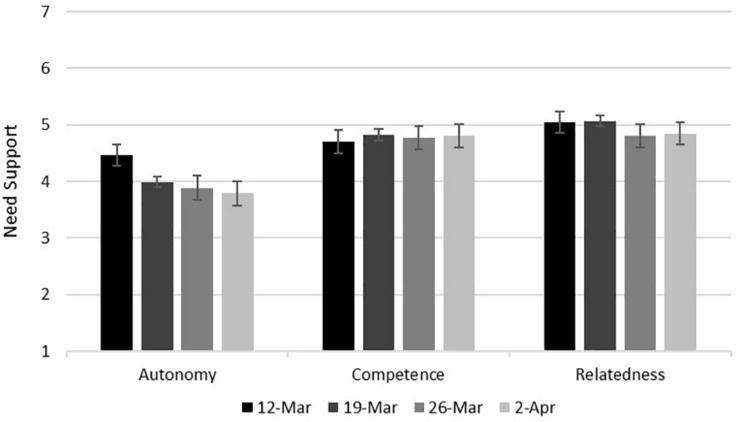
Need support in responding to COVID-19 as a function of the day of the study. Error bars represent 95% confidence intervals. Scale midpoint is 4.

As shown in Table 7 and summarized in Figure 2, autonomy was the only type of need support that showed significant differences across the 4 Thursdays of the study. Participants reported significantly less autonomy support in responding to COVID-19 after March 12 than they did on March 12. Autonomy support in responding to COVID-19 on March 19, March 26, and April 2 did not differ significantly. The Bonferoni-adjusted *p*-value within each of the six *post-hoc* comparisons for this ANOVA was 0.008, and each significant *post-hoc* test surpassed this criterion.

**TABLE 7:** Tests of differences between the days of the study.

Measure and test	*df*s	*F*	*P*	η^2^	Mean diff.	Sig.	95% CI
**Autonomy**	**(3, 397)**	**8.64**	<**0.001**	**0.06**			
**March 12-March 19**					**−0.46**	**0.007**	**[−0.83, −0.10]**
**March 12-March 26**					**−0.0.57**	<**0.001**	**[−0.93, −0.20]**
**March 12-April 2**					**−0.67**	<**0.001**	**[−1.03, −0.30]**
March 19-March 26					−0.10	0.886	[−0.47, 0.26]
March 19-April 2					−0.20	0.479	[−0.57, 0.16]
March 26-April 2					−0.10	0.896	[−0.46, 0.27]
Competence	(3, 397)	0.28	0.840	0.00			
March 12-March 19					0.13	0.828	[−0.26, 0.52]
March 12-March 26					0.07	0.971	[−0.32, 0.45]
March 12-April 2					0.11	0.896	[−0.28, 0.49]
March 19-March 26					−0.06	0.977	[−0.45, 0.33]
March 19-April 2					−0.02	0.999	[−0.41, 0.36]
March 26-April 2					0.04	0.994	[−0.35, 0.43]
Relatedness	(3, 397)	1.60	0.189	0.01			
March 12-March 19					0.03	0.995	[−0.34, 0.41]
March 12-March 26					−0.22	0.421	[−0.60, 0.15]
March 12-April 2					−0.19	0.567	[−0.57, 0.19]
March 19-March 26					−0.26	0.291	[−0.64, 0.12]
March 19-April 2					−0.23	0.418	[−0.60, 0.15]
March 26-April 2					0.03	0.995	[−0.34, 0.41]
Promotion	(3, 397)	1.50	0.214	0.01			
March 12-March 19					−0.10	0.949	[−0.55, 0.36]
March 12-March 26					0.11	0.924	[−0.34, 0.56]
March 12-April 2					0.26	0.454	[−0.19, 0.71]
March 19-March 26					0.21	0.649	[−0.25, 0.66]
March 19-April 2					0.35	0.184	[−0.10, 0.81]
March 26-April 2					0.15	0.832	[−0.30, 0.60]
**Prevention**	**(3, 397)**	**7.06**	<**0.001**	**0.05**			
**March 12-March 19**					**0.49**	<**0.001**	**[0.18, 0.79]**
March 12-March 26					0.12	0.741	[−0.19, 0.43]
**March 12-April 2**					**0.36**	**0.011**	**[0.06, 0.67]**
**March 19-March 26**					**−0.37**	**0.011**	**[−0.67, −0.06]**
March 19-April 2					−0.12	0.733	[−0.43, 0.18]
March 26-April 2					0.24	0.163	[−0.06, 0.55]

*Tukey post-hoc tests. Positive numbers indicate higher means for the second condition within the pair. CI, confidence interval. Bold font indicates rows with significant effects.*

Whereas the judged usefulness of promotion for responding to COVID-19 did not differ significantly across the days of the study, the judged usefulness of prevention for responding to COVID-19 went up and down across the days of the study. Table 7 shows these results and Figure 1 summarizes them. The Bonferoni-adjusted *p*-value within each of the six *post-hoc* comparisons for the ANOVA on prevention usefulness was 0.008, and only the significant increase between March 12 and March 19 surpassed this criterion.^9^

## Discussion

The primary purpose of this study was to examine the judged usefulness of promotion and prevention focus for responding to COVID-19 in the first 4 Thursdays of the pandemic. We developed a new judged usefulness measure for this research, and the items corresponded well to promotion and prevention factors. As expected, participants judged prevention focus to be more useful than promotion focus for responding to COVID-19. Because many people who can spread COVID-19 are asymptomatic, and the consequences of contracting the virus can be dire (Furukawa et al., 2020), prevention focus appears to be adaptive for responding to this virus (Centers for Disease Control and Prevention, 2020c).^10^ This study used the new measure to test hypotheses about how psychological need support related to judged usefulness of promotion and prevention focus for responding to COVID-19. It also explored day-to-day differences in judged usefulness, need support, and actions taken to respond to the virus.

We based hypotheses about relationships between need support and regulatory focus on earlier research on the need-support model (Vaughn, 2017). This model bridges regulatory focus theory (Higgins, 1997, 1998) and self-determination theory (Deci and Ryan, 2000; Ryan and Deci, 2017) by proposing how regulatory focus and psychological need support can influence each other. As anticipated, in responding to COVID-19, participants’ autonomy support related positively to the judged usefulness promotion focus. These results conceptually replicate findings of earlier research on recalled everyday types of experiences (Vaughn, 2017, 2019; Kim et al., 2019; Vaughn et al., 2020) and experimental performance tasks (Vaughn, 2017). Additionally, they complement the positive relationships research has found between promotion focus and autonomous, “want-to” motivation (Lalot et al., 2018; Vaughn, 2018; Laroche et al., 2019).

The results for prevention did not support our hypotheses. In responding to COVID-19, participants’ relatedness support associated positively (not negatively) to the judged usefulness of prevention focus. Additionally, when controlling for relationships among the types of need support, relatedness was the only one that associated significantly with the judged usefulness of prevention focus. In earlier research (Vaughn, 2017, 2019; Vaughn et al., 2020) prevention focus related positively to competence support and negatively to autonomy and relatedness support. Participants in these earlier studies often described everyday experiences where they fulfilled duties and obligations to people with whom they did not feel particularly close (also see Vaughn, 2018). In contrast, participants in the current study who felt closer to others may have judged that if they made mistakes in responding to COVID-19, these other people would suffer more for it.

It appears that prevention focus in responding to COVID-19 was also enthusiastic. We had expected that the enthusiasm item would only load on the promotion factor (consistent with Ouschan et al., 2007), but it loaded on both promotion and prevention factors. The enthusiasm item also related positively to relatedness support (like prevention usefulness) and autonomy support (like promotion usefulness). If participants’ responses to COVID-19 were often to protect others about whom they cared most, prevention focus in this context could be energetic and personally meaningful. Indeed, research shows that relatedness support associates positively with meaning in life (Hicks and King, 2009; Lambert et al., 2013; Martela et al., 2018), and prosocial behavior can enhance well-being and subjective vitality (Martela and Ryan, 2016). Future research could examine whether prevention focus generally is more enthusiastic when people are protecting those about whom they care deeply.

### *Post-hoc* Hypotheses About Differences Between Days of the Study

This research sampled a different group of 100–101 participants on each of the first 4 Thursdays of the pandemic, and we discuss the following results in the interest of transparency and hypothesis generation. This study confounds sample with time period, and thus all our *post-hoc* hypotheses about differences between days of the study are tentative.

The observed relationships between need support and usefulness judgments were stronger on all 3 Thursdays after March 12 than on March 12. This finding could indicate that the questions about need support and regulatory focus in responding to COVID-19 were less meaningful to participants on March 12. On March 12, relatively few participants had experienced direct consequences of the pandemic, as shown by actions they had already taken to respond to COVID-19. Table 6 shows that the number of participants who reported having taken such actions increased dramatically from March 12 to March 19 and stayed high after that. The sharp increase in actions taken to respond to COVID-19 corresponded to the beginning of widespread states of emergency, shown in Table 8.

**TABLE 8:** Timeline of selected COVID-19 events.

Dates	Events
Jan. 23	• Chinese authorities place the city of Wuhan on lockdown to slow the spread of the to-be-named coronavirus.
March 8	• Italy places all its residents on lockdown to slow the spread of COVID-19.
March 11	• The World Health Organization declares COVID-19 a pandemic. The number of diagnosed COVID-19 cases is 116 in Canada and 1,205 in the United States.
March 12	• **First day of data collection** Quebec is the first province to declare a state of emergency.
March 13	• The United States declares a national emergency. Canada’s Parliament unanimously agrees to close for 5 weeks to slow the spread.
March 15	• The White House issues guidelines on how to avoid spreading the virus, which include avoiding gatherings of more than 10 people for the **next 15 days**.
March 18	• Nine Canadian provinces and territories have declared states of emergency. Unemployment has skyrocketed in Canada and the United States since the previous week. The number of diagnosed COVID-19 cases is 727 in Canada and 8,074 in the United States.
March 19	• **Second day of data collection** California is the first state to issue a statewide stay-at-home order.
March 22	• All 12 Canadian provinces and territories have declared states of emergency.
March 25	• Unemployment has continued to skyrocket in Canada and the United States. The number of diagnosed COVID-19 cases is 3,409 in Canada and 64,916 in the United States.
March 26	• **Third day of data collection** Twenty-two states have issued statewide stay-at-home orders.
March 28	• The U.S. Centers for Disease Control and Prevention urges residents of New York, New Jersey, and Connecticut to refrain from all non-essential travel for 2 weeks.
March 29	• The White House extends social distancing guidelines **through April 30**.
April 1	• In a press conference, Prime Minister Trudeau says that the need to stay at home will **continue for weeks** in Canada. Unemployment has continued to skyrocket in Canada and the United States. The number of diagnosed COVID-19 cases is 9,731 in Canada and 212,747 in the United States.
April 2	• **Final day of data collection** Thirty-nine states have issued statewide stay-at-home orders.

*Bold font indicates messages from heads of state about the likely duration of stay-at-home orders. The Supplementary Material contains the references for this table and indicates which sources informed which entries in the table.*

Autonomy support in responding to COVID-19 dropped significantly from March 12 to March 19 and stayed lower after that. This finding suggests that participants felt less able to do what they really wanted in responding to COVID-19 after widespread stay-at-home orders had started. Competence support and relatedness support in responding to COVID-19 remained stable and high over the Thursdays of the study. Other psychological research on COVID-19 that used the same measure of relatedness support found no significant decrease in relatedness support among Prolific participants in the United States and United Kingdom who were sampled on February 12, 2020, and again April 1–9, 2020 (Folk et al., 2020). Additionally, a representative sample of Americans studied in late January/early February 2020, in late March 2020, and late April 2020 showed no significant change in loneliness (Luchetti et al., 2020). These and the current findings suggest that people found ways to feel competent and connected to others in responding to the pandemic.

Day-to-day variation in the judged usefulness of promotion and prevention focus did not correspond to day-to-day variation in need support: promotion stayed low (unlike autonomy support), and prevention went up and down (unlike relatedness support). These results on the prevention measure do not correspond to any variables in the current study. However, they do correspond to messages from heads of state about the likely duration of stay-at-home orders. We note these messages in bold font in Table 8. On March 12, there were no widespread shutdowns in the United States or Canada, and judged usefulness of prevention focus was low. Between March 12 and March 19, states of emergency were declared, and widespread stay-at-home orders began. Symptoms of COVID-19 appear within 2 weeks (Lauer et al., 2020). If participants on March 26 expected to be able to relax their caution in another week, it could explain the small decline in judged usefulness of prevention focus between March 19 and March 26. By April 1, however, leaders had communicated that states of emergency and stay-at-home orders would need to continue for weeks longer, which may explain the small rise in judged usefulness of prevention focus between March 26 and April 2.

### Implications for Persuasive Messaging About COVID-19

If people generally perceive enthusiastic prevention focus in the service of protecting loved ones to be useful for responding to COVID-19, the current findings could inform persuasive messaging for responding to the virus. Regulatory fit (Higgins, 2000) occurs when the strategies one considers for pursuing a goal (e.g., exerting self-control and being careful) fit and sustain one’s regulatory focus toward the goal (e.g., protecting loved ones). Regulatory fit feels right and can be motivating (e.g., Freitas and Higgins, 2002) because people can attribute this feeling of rightness to what they are judging (e.g., Vaughn et al., 2006a, b, 2010b). They may assume that if they feel right when thinking about something (e.g., wearing a mask), it is because what they are thinking about is right. Regulatory fit can enhance persuasion through advocacy messages, which have explicit intent to persuade (e.g., Cesario et al., 2004, 2008; Lee and Aaker, 2004; Koenig et al., 2009; Ludolph and Schulz, 2015), and through narratives, where the persuasive intent is more subtle (e.g., Vaughn et al., 2009, 2010a).

### Limitations

This study has longitudinal aspects, because it sampled 100 people on the first 4 Thursdays of the pandemic. However, it did not follow individual people across 4 weeks, so it does not assess individual-level change. Thus, differences between who chose to participate on different days of the study could have contributed to the differences in results between days of the study. Future research on responses to COVID-19 could take a fully longitudinal approach.

This research did not have a representative sample of Americans and Canadians. Prolific and MTurk samples are similar (Peer et al., 2017), and MTurk samples are not representative of the general U.S. population (Goodman et al., 2013; Walters et al., 2018). For example, MTurk samples tend to be younger, more educated, less employed, have more White and Asian respondents and fewer Black or African-American and Latinx or Hispanic respondents than the general U.S. population (Walters et al., 2018). COVID-19 has stronger impacts on people who are older (McCarthy, 2020) and on people of color (Centers for Disease Control and Prevention, 2020a). Our study probably under-represented groups that were hit hardest by COVID-19, and a representative sample could show stronger results.

Replications at different points in time could find different results, because of changes in policies and attitudes about social distancing and other mitigation responses. Research suggests that political attitudes (e.g., Reves, 2020) and attentiveness to COVID-related news and COVID-19-related attitudes and beliefs (Oosterhoff and Palmer, 2020; Pedersen and Favero, 2020) relate strongly to attitudes about social distancing. If “quarantine fatigue” (Rogers, 2020) and “mask rage” (Garcia-Roberts, 2020) become more common as the pandemic continues, the predominance of prevention over promotion in responding to COVID-19 could lessen. Additionally, if protecting others against the virus comes to feel more like a pressuring duty and obligation, the relationships between judged usefulness of prevention focus and autonomy and relatedness support in responding to COVID-19 could turn negative.

Finally, cultural context could influence the judged usefulness of promotion and prevention focus for responding to COVID-19. The current study’s participants resided in the U.S. and Canada, which are individualist cultures where people tend to be somewhat promotion-oriented (e.g., Lee et al., 2000). In relatively collectivist cultures, which emphasize duties and obligations (e.g., Miller et al., 2011; Buchtel et al., 2018), people could be even more likely than those in the current research to judge prevention focus more useful than promotion focus for responding to COVID-19.

## Conclusion

COVID-19 is such an urgent threat that an understandable reaction could be to assume that psychological research pertaining to it should be directly applicable to saving lives. For research on goals and motivation, that could mean assuming all research pertaining to COVID-19 should be about how persuasive communications could stop or slow the spread. This area of research is growing (e.g., Luttrell and Petty, 2020; Pfattheicher et al., 2020). However, to have a good intervention based on regulatory focus and psychological need support, one first needs good measures and a good understanding of how people tend to view the problem. These were goals of the current research. We found that judged usefulness of promotion and prevention focus is a construct that can be measured in the context of responding to COVID-19, and as expected, participants judged prevention to be more useful than promotion for responding to the virus. We also found that “Being enthusiastic,” which is an item we had expected would load on the judged usefulness of promotion factor, also loaded on the judged usefulness of prevention factor. Enthusiasm as an aspect of the judged usefulness of prevention focus has not been found before in published research. The current findings suggest that many actions taken to respond to COVID-19 are in the service of protecting others, and that these responsibilities are more deeply meaningful and enjoyable to pursue the closer and more connected one feels to others. Overall, this study suggests that messages emphasizing social connection could be especially persuasive for responding to COVID-19, given the judged usefulness of prevention for responding to the virus. We hope future research will explore this possibility.

## Data Availability Statement

The datasets generated for this study can be found in the online repositories. The names of the repository/repositories and accession number(s) can be found below: Open Science Framework: https://osf.io/8ek2w.

## Ethics Statement

The studies involving human participants were reviewed and approved by the Institutional Review Board of Ithaca College. The patients/participants provided their written informed consent to participate in this study.

## Author Contributions

LV conceptualized and designed the research, acquired the data, and wrote the first draft of the manuscript. LV, CG, and RC contributed to the analysis and interpretation of the data, commented on previous versions of the manuscript, read and approved the final manuscript. CG and RC authorship order was decided by a coin flip. All authors contributed to the article and approved the submitted version.

## Conflict of Interest

The authors declare that the research was conducted in the absence of any commercial or financial relationships that could be construed as a potential conflict of interest.

## Footnotes

1 No state, province, or territory had a majority of participants. Ontario had the most with 61 participants (15.2%), followed by Florida with 23 (5.6%), Texas with 22 (5.4%), New York with 18 (4.4%), and Pennsylvania with 13 (3.2%).

2 G^∗^Power does not include factor analysis. However, 200 participants are enough to support an exploratory factor analysis under moderately good conditions (communalities of 0.40–0.70, with at least three measured variables per theorized factor; Fabrigar and Wegener, 2012). Several of the communalities in our analyses were slightly below this range, and 400 participants can support an exploratory factor analysis under these conditions (Fabrigar and Wegener, 2012).

3 The table order is determined by the best order for the “Results” section.

4 We did sensitivity power analyses for bivariate normal correlations. According to G^∗^Power, 401 participants provide 80% power to detect a Pearson *r* of 0.14, *p* = 0.05, two-tailed. One hundred participants provide 80% power to detect a Pearson *r* of 0.28, *p* = 0.05, two-tailed.

5 According to G^∗^Power, 401 participants provide 80% power to detect a difference in a paired-samples t-test of *d* = 0.14, *p* = 0.05, two-tailed, and 100 participants provide 80% power to detect a difference in a paired-samples *t*-test of *d* = 0.28, *p* = 0.05, two-tailed.

6 We did not expect that the other regulatory focus would be a significant predictor in the multiple regression analyses because the factor analysis revealed that the promotion and prevention factors were not strongly related. However, as in other research (Higgins et al., 2001; Camacho et al., 2003; Vaughn, 2017), we controlled for the other regulatory focus in the regression analyses.

7 We did sensitivity power analyses for single regression coefficients in four-predictor linear multiple regressions with 401 participants and with 100 participants. G^∗^Power provides *f*^2^s for this type of analysis. In our multiple regressions, we used *sr*^2^ as the measure of effect size, as recommended by Disabato (2016). The variable, *sr*^2^, is the correlation between the predictor of interest and the dependent variable, controlling for the relationships between the other predictors and the predictor of interest. To translate between *f*^2^ and *sr*^2^, we used an online calculator (Lenhard and Lenhard, 2016) to determine the correlation *r*s that were equivalent to the square roots of the *f*^2^s, and we squared those *r*s. According to G^∗^Power, 401 participants provide 80% power to detect an individual coefficient in a four-predictor multiple regression with *f*^2^ = 0.02, *p* = 0.05, two-tailed, which is equivalent to *sr*^2^ of 0.02. Additionally, 100 participants provide 80% power to detect an individual coefficient in a four-predictor multiple regression with *f*^2^ = 0.08, *p* = 0.05, two-tailed, which is equivalent to *sr*^2^ of 0.07.

8 According to G^∗^Power, 401 participants provide 80% power to detect a critical chi-square value of 7.814, *p* = 0.05, two-tailed, in an analysis with three degrees of freedom. We also did a power analysis with the Bonferoni-adjusted *p*-value, because of the large number of exploratory tests on these items. With the adjusted *p*-value, this design provides 80% power to detect a critical chi-square value of 14.320.

9 We did sensitivity power analyses for four-group, one-way ANOVAs. G^∗^Power provides *f*s for this type of analysis. In our ANOVAs, we used η^2^ as the measure of effect size. We used an online calculator (Lenhard and Lenhard, 2016) to translate between *f* and η^2^. According to G^∗^Power, 401 participants provide 80% power to detect an omnibus effect in a one-way ANOVA with *f* = 0.17, *p* = 0.05, two-tailed, which is equivalent to η^2^ of 0.03.

10 Prevention focus and promotion focus both are self-regulatory orientations for approaching pleasure and avoiding pain (Higgins, 1997, 1998). When prevention focused, people are motivated to approach non-losses and avoid losses (e.g., protect friends and family from COVID-19 vs. expose them to the virus), and when promotion focused, people are motivated to approach gains and avoid non-gains (e.g., explore eating inside a new restaurant vs. miss out on this opportunity). The effectiveness of each regulatory focus depends on how well one’s focus fits one’s circumstances (e.g., Higgins, 1997, 1998, 2000, 2005; Molden et al., 2007; Scholer and Higgins, 2012; Scholer et al., 2019a). A common misunderstanding of regulatory focus theory is that prevention focus is the same as having an avoidance goal (e.g., Molden et al., 2007; Scholer and Higgins, 2008, 2013; Summerville and Roese, 2008). In performance settings, avoidance motivation and avoidance goals are less effective than approach motivation and approach goals (e.g., Elliot and Church, 1997; Elliot, 2006). However, the avoidance strategies that fit prevention-focused goals are at a lower level of the goal-pursuit hierarchy than goals are, and they are not the same as prevention-focused goals (Scholer and Higgins, 2008, 2013; Scholer et al., 2019b).
