# Supplementary material for: Need Support and Regulatory Focus in Responding to COVID-19
Source: Front Psychol. 2020 Nov 19;11:589446. doi: 10.3389/fpsyg.2020.589446 (PMC7717948; doi:10.3389/fpsyg.2020.589446)
Supplement: Supplementary file 1 [file Table_1.DOCX]

Methodology Reporting

In this document, a dashed line indicates the location of a page break in the online questionnaire.

-------------------------------------------------------------------------------------------------------------------------------

Before you start, please switch off phone/ e-mail/ music so you can focus on this study.

Thank you!

Please enter your Prolific ID here:

---------------------------------------------------------------------------------------------------------------------

Your Personal Responses to the COVID-19 Pandemic

First, we would like to learn about how you personally are responding to the COVID-19 coronavirus pandemic. This is a general question, and you can write about your thoughts, feelings, and/or behaviors.

Please take a minute or two and write about your responses to this pandemic.

---------------------------------------------------------------------------------------------------------------------

Your Personal Responses to the COVID-19 Pandemic

Earlier, you wrote:

[piped in what they wrote on the previous page]

Please indicate how much you agree with the following statements about your responses to the COVID-19 pandemic.

(1 = strongly disagree; 4 = neither agree nor disagree; 7 = strongly agree)

- I am free to do things my own way.
- I successfully complete difficult tasks and projects.
- I feel a sense of contact with people who care for me, and whom I care for.
- I have a lot of pressures I could do without.
- I experience some kind of failure, or am unable to do well at something.
- I am lonely.
- My choices express my “true self.”
- I take on and master hard challenges.
- I feel close and connected with other people who are important to me.
- There are people telling me what I have to do.
- I do stupid things, that make me feel incompetent.
- I feel unappreciated by one or more important people.
- I am really doing what interests me.
- I do well even at the hard things.
- I feel a strong sense of intimacy with the people I spend time with.
- I have to do things against my will.
- I struggle doing something I should be good at.
- I have disagreements or conflicts with people I usually get along with.

---------------------------------------------------------------------------------------------------------------------

Your Personal Responses to the COVID-19 Pandemic

Earlier, you wrote:

[piped in what they wrote on the earlier page]

Please indicate how much each of the following would support or impair how you respond to the COVID-19 pandemic.

(1 = strongly impair, 2, 3, 4 = neither impair nor support, 5, 6, 7 = strongly support)

- Not making mistakes
- Trying new things just because they could be interesting
- Exerting self-control
- Being enthusiastic
- Fulfilling my duties and obligations
- Being spontaneous
- Doing what is expected of me
- Not missing out on anything good
- Being careful
- Doing what I would ideally like to

---------------------------------------------------------------------------------------------------------------------

Your Personal Responses to the COVID-19 Pandemic

Please indicate which of the following actions you have already taken.

(0 = no, 1 = yes)

- Buying soap and disinfectants
- Stocking up on groceries
- Stocking up on medicine
- Checking in with work and school about closures
- Paying attention to local news
- Figuring out how to work from home
- Washing your hands regularly
- Reaching out to others for support
- Providing support to others
- Self-quarantining
- Talking with your neighbors about emergency planning
- Creating an emergency contact list
- Keeping track of school dismissals in your community
- Not gathering in public places
- Talking with supervisors or teachers about work that can be done from home
- Identifying aid organizations in your community
- Staying away from others who are sick
- Limiting close contact with others (about 6 feet)
- Cleaning frequently touched surfaces and objects daily with household detergent and water
- Covering your coughs and sneezes with a tissue

---------------------------------------------------------------------------------------------------------------------

Your Personal Responses to the COVID-19 Pandemic

Have there been any COVID-19 cases reported in the state or province where you live?

(1 = no, 2 = unsure, 3 = yes)

Have there been any COVID-19 cases reported in the city or town where you live?

(1 = no, 2 = unsure, 3 = yes)

Do you personally know anyone who has been diagnosed with COVID-19?

(1 = no, 2 = unsure, 3 = yes)

[On March 26, we added this question:

If yes to the previous question]

How many people do you personally know who have been diagnosed with COVID-19? (Please just give a number, e.g., 2.)

---------------------------------------------------------------------------------------------------------------------

Demographic Questions

How old are you? Please write only a number, for example, 18.

What gender are you? male female other prefer not to answer

What is your ethnic group? (you may select more than one)

Asian

Black or African-American

Hispanic or Latino

Multiethnic

Native American or Alaska Native

Native Hawaiian or Pacific Islander

White

Other

What is your nation of birth?

[From the question above, we coded another numeric variable: country of residence]

What language was spoken in the home where you grew up?

Is English your first language? Yes No

[On March 26, we added the following two questions:]

In which state, province, or territory do you live?

Where do you currently reside? [drop-down list of states, provinces, and territories]

[To get approximate data on these questions for the first 200 participants, we coded for location based on the latitude/longitude information that Qualtrics automatically provides. Qualtrics estimates latitude and longitude from IP addresses by comparing them to a location database. According to Qualtrics, these estimates typically are accurate to the city level inside the US and to the country level outside the US. However, IP addresses can show the location of the coffee shop or airport where the participant did the survey, or the location of the internet service provider’s exit point for online service. Some latitude/longitude data in the first two weeks suggest that a few participants were in other countries when they took the survey – possibly in airports or other travel locations.]

What is the highest level of education you have completed?

What is your primary occupation?

-----------------------------------------------------------------------------------------------------------------------------

Impressions of the Study

Thank you for participating!

What are your impressions of the study?

Were you distracted by anything going on in the room while you were doing the study? (yes/no)

--------------

If yes, participants went to a page that asked them what they were distracted by and how distracted they were.

**References**

Vaughn, L. A. (2017a). Data from foundational tests of the need-support model: A framework

for bridging regulatory focus theory and self-determination theory. *Journal of Open Psychology Data*, *5*, 2. <https://doi.org/10.5334/jopd.30>

Vaughn, L. A. (2017b). Foundational tests of the need-support model: A framework for bridging

regulatory focus theory and self-determination theory. *Personality and Social Psychology Bulletin, 43,* 313-328. <https://doi.org/10.1177/0146167216684132>
