# Supplementary material for: Need Support and Regulatory Focus in Responding to COVID-19
Source: Front Psychol. 2020 Nov 19;11:589446. doi: 10.3389/fpsyg.2020.589446 (PMC7717948; doi:10.3389/fpsyg.2020.589446)
Supplement: Supplementary file 2 [file Table_2.DOCX]

Supplementary Table

Need Support and Regulatory Focus in Responding to COVID-19

Leigh Ann Vaughn, Chase A. Garvey, and Rachael D. Chalachan

Ithaca College

*The second and third authors contributed equally, and authorship was decided by a coin flip.

*References for Table 1 – Timeline of Selected COVID-19 Events*

| Reference | Information in Table 1 |
| --- | --- |
| COVID-19 pandemic in Canada. (2020, June 30). In *Wikipedia*. <https://en.wikipedia.org/wiki/COVID-19_pandemic_in_Canada> | - Dates that states of emergency were issued, and number of cases by day |
| Hauck, G., Gelles, K., Bravo, V., & Thorson, M. (2020, May 27). Four months in: A timeline of how COVID-19 has unfolded in the US. USA Today. <https://www.usatoday.com/in-depth/news/nation/2020/04/21/coronavirus-updates-how-covid-19-unfolded-u-s-timeline/2990956001/> | - Timeline of events |
| Rev. (2020, April 1). Justin Trudeau Canada COVID-19 Press Conference Transcript April 1. <https://www.rev.com/blog/transcripts/justin-trudeau-canada-covid-19-press-conference-transcript-april-1> | - Transcript of Trudeau’s April 1 press conference |
| Mendelson, L. (2020, May 20). *Stay on top of “stay at home” – A list of statewide orders*. Littler. <https://www.littler.com/publication-press/publication/stay-top-stay-home-list-statewide> | - Dates that states issued stay-at-home orders |
| Secon, H., Woodward, A., & Mosher, D. (2020, June 30). *A comprehensive timeline of the coronavirus pandemic at 6 months, from China's first case to the present.* Business Insider. <https://www.businessinsider.com/coronavirus-pandemic-timeline-history-major-events-2020-3> | - Date that Italy locked down |
| Statistics Canada. (2020, April 9). *Labour force survey, March 2020.* <https://www150.statcan.gc.ca/n1/daily-quotidien/200508/dq200508a-eng.htm> | - Surge in unemployment in Canada |
| Timeline of the COVID-19 pandemic in Canada. (2020, June 30). In *Wikipedia*. [https://en.wikipedia.org/wiki/Timeline_of_the COVID-19_pandemic_in_Canada](https://en.wikipedia.org/wiki/Timeline_of_the%20COVID-19_pandemic_in_Canada) | - Timeline of events |
| Timeline of the COVID-19 pandemic in the United States. (2020, June 30). In *Wikipedia*. <https://en.wikipedia.org/wiki/Timeline_of_the_COVID-19_pandemic_in_the_United_States> | - Timeline of events, including number of cases per day |
| United States Department of Labor. (2020, April 2). *Unemployment insurance weekly claims report.* <https://www.dol.gov/sites/dolgov/files/OPA/newsreleases/ui-claims/20200551.pdf?utm_source=link_newsv9&utm_campaign=item_308276&utm_medium=copy> | - Surge in unemployment in the United States |
| World Health Organization. (2020, March 11). *WHO Director-General's opening remarks at the media briefing on COVID-19 - 11 March 2020*. <https://www.who.int/dg/speeches/detail/who-director-general-s-opening-remarks-at-the-media-briefing-on-covid-19---11-march-2020> | - WHO declaration of the pandemic |
